# Two forms of one complication: Late erosive and nonerosive postpancreatectomy hemorrhage following laparoscopic pancreaticoduodenectomy

**DOI:** 10.1097/MD.0000000000016394

**Published:** 2019-07-26

**Authors:** Feng Feng, Xuehui Cao, Xueqing Liu, Jianzhang Qin, Zhongqiang Xing, Jiayue Duan, Chen Liu, Jianhua Liu

**Affiliations:** a Second Hospital of Hebei Medical University, Shijiazhuang; b Fudan University Shanghai Cancer Center, Shanghai, China.

**Keywords:** erosive factors, laparoscopic pancreaticoduodenectomy, postpancreatectomy hemorrhage

## Abstract

Postpancreatectomy hemorrhage (PPH) remains a rare but lethal complication following laparoscopic pancreaticoduodenectomy (LPD) in the modern era of advanced surgical techniques. The main reason for early PPH (within 24 hours following surgery) has been found to be a failure of hemostasis during the surgical procedure. The reasons for late PPH tend to be variate. Positive associations have been identified between late PPH and intraabdominal erosive factors such as postoperative pancreatic fistula, bile leakage, gastrointestinal fistula, and intraabdominal infection. Still, some patients suffer PPH who do not have these erosive factors. The severity of bleeding and clinical prognosis of erosive and nonerosive PPH following LPD is different.

We analyzed the electronic clinical records of 33 consecutive patients undergoing LPD and experiencing one or more episodes of hemorrhage after postoperative day 1 in this study. All patients received an LPD with standard lymphadenectomy. The patient's hemorrhage-related information was extracted, such as interval from surgery to bleeding, presentation, bleeding site, severity, management, and clinical prognosis. Based on our clinical practice, we proposed a treatment strategy for these 2 forms of late PPH following LPD.

Of these 33 patients, 8 patients (24.24%) developed nonerosive bleeding, and other 25 patients (75.76%) suffered from postoperative hemorrhage caused by various intraabdominal erosive factors. The median interval from the LPD surgery to postoperative hemorrhage for both groups was 11 days, and no significant differences were found (*P* = .387). For patients with erosive bleeding, most (60%) underwent their episodes of bleeding on postoperative days 5 to 14. For patients with nonerosive bleeding, most (75%) began postoperative hemorrhage 2 weeks after surgery, and 50% of these patients had bleeding between postoperative days 20 and 30. In the present study, 64% (16/25) of patients with erosive bleeding and 87.5% (7/8) of patients with nonerosive bleeding had internal bleeding. The fact that 90% (9/10) of all gastrointestinal bleeding patients had intraabdominal erosive factors indicated strong relationships between gastrointestinal hemorrhage and these erosive factors. The bleeding sites were detected in most patients, except for 4 patients who received conservative treatments. For patients with erosive bleeding, the most common bleeding site detected was the pancreatic remnant (43.48%); others included the hepatic artery (39.13%), splenic artery (13.04%), and left gastric artery (4.35%). For patients with nonerosive bleeding, the most common bleeding site was the hepatic artery (83.33%), and the 2nd most frequent site was the splenic artery (16.67%). No hemorrhage from pancreaticojejunal anastomosis occurred in the patients with nonerosive bleeding. Statistical significance was noted between these 2 groups in hemorrhage severity (*P* = .012), management strategies (*P* = .001), rebleeding occurrence (*P* = .031), and prognosis outcome (*P* = .010). The patients with intraabdominal erosive factors tended to have a higher risk of grade C bleeding (68.00%) than that of their nonerosive bleeding counterparts (12.50%). As for treatment strategy for postoperative bleeding, the favorable method to manage nonerosive bleeding was conservative and endovascular treatments if the patients’ hemodynamics was stable. All these nonerosive bleeding patients survived. On the contrary, 22 patients (88.00%) in the erosive bleeding group had a 2nd surgical procedure, and the mortality was 56.00%. In this group, 2 patients received conservative therapy due to the demand of their family and expired. One patient underwent endovascular treatment and had another episode of hemorrhage, finally dying from multi-organ failure. No patients in the nonerosive bleeding group suffered from rebleeding after complete hemostasis, and 44.00% of patients with erosive bleeding underwent a 2nd episode of postoperative bleeding.

Erosive and nonerosive PPH are 2 forms of this lethal complication following LPD. Their severity of bleeding, rebleeding rate, and treatment strategy are different. Patients with erosive factors tend to have a higher incidence of grade C bleeding, rebleeding, and mortality. Factors influencing treatment protocols for PPH include the existence of intraabdominal erosive factors, patient hemodynamics, possibility to detect the bleeding site during endovascular treatment, and surgeon's preference. The performance of endovascular treatment with stent repair for managing postoperative hemorrhage after LPD depends on the discovery of the bleeding site. Surgery should be reserved as an emergent and final choice to manage PPH.

## 1 Introduction

Although great advances have been made in pancreatic surgical techniques, postpancreatectomy hemorrhage (PPH) remains a severe postoperative complication after laparoscopic pancreaticoduodenectomy (LPD) with low incidence but general lethality. According to previous reports, hemorrhage occurs in 3% to 16% of patients undergoing pancreatectomy, with accompanying mortality rates between 11% and 54%, and is responsible for the majority of deaths following LPD.^[1]^

In accordance with the International Study Group of Pancreatic Surgery definition, based on the time of onset, PPH was categorized into early hemorrhage (<24 hours following the operation), which is generally regarded as a failure of the surgical procedure, and late (>24 hours following the operation), with diverse reasons.^[2]^ In this study, we focus only on late hemorrhage and do not involve those early bleeding cases mainly due to its explicit cause and subsequent treatment protocols. Several researchers have demonstrated the association between this deadly complication and intraabdominal erosive factors such as postoperative pancreatic fistula (POPF), bile leakage, gastrointestinal fistula, and abscess.^[3]^ Incomplete pancreatic resections and POPF are considered major potential risk factors, accounting for PPH by numerical pancreatic specialists.^[1]^ In addition, the risks for postoperative bleeding may increase, secondary to an abdominal abscess, which causes inflammatory responses, tissue edema, and, ultimately, injury to the vascular integrity. However, not all patients suffering from late PPH had previously existing intraabdominal erosive factors. Wellner et al reported on 22 grade C PPHs out of 1082 pancreatic resections, and 7 patients’ cases in this group were not associated with POPF.^[4]^ In 2010, Lee et al reported that 27 out of 907 patients underwent PPH following pancreaticoduodenectomy (PD), but POPF was observed in only 12 cases (44.4%).^[5]^ One possible explanation for nonerosive PPH could be pseudoaneurysm formation following surgical injury to the blood vessels and secondary rupture due to a sudden increase of blood pressure. No previous studies report nonerosive PPH; however, in our own experience, erosive PPHs related to POPF, bile leakage, gastrointestinal fistula, and abscess are always accompanied by relatively high rebleeding rates and mortality, compared to their nonerosive counterparts. Different underlying etiologies may exist, because not all late PPH appears to be equal. We believe that late PPH can be classified into 2 types, including erosive PPH and nonerosive PPH, with different treatment protocols and clinical outcomes. The aim of the present study was to analyze PPH-related parameters and provide separate treatment strategies for erosive and nonerosive PPH.

## 2 Patients and methods

From January 2013 to July 2018, 440 patients received LPD at our department; 33 consecutive patients undergoing LPD and experiencing one or more episodes of hemorrhage after postoperative day 1 were included in this study. This clinical database was maintained prospectively and analyzed in the postoperative period. All patients involved in this study gave their informed consent. Institutional review board approval of our hospital was also obtained for this study. Patient characteristics; definitions of the pancreatic leak, bile leak, and intraabdominal abscess; onset, location, and severity of postoperative hemorrhage; treatment protocol; and mortality were extracted and evaluated. Of all 33 patients, 8 patients (24.24%) developed nonerosive bleeding, and the other 25 patients (75.76%) developed postoperative hemorrhage for several reasons, such as pancreatic fistula, bile leakage, and intraabdominal abscess. This patient cohort included 21 males and 12 females, whose ages ranged from 33 to 79 years (mean 59.24 ± 11.44 years). The indications for LPD included pancreatic head cancer in 9 patients, distal cholangiocarcinoma in 12 patients, and duodenal adenocarcinoma in 12 patients. All patients received an LPD with standard lymphadenectomy. The gastrointestinal tract reconstruction was performed according to Child's anastomosis. The pancreatic remnant, biliary stump, and stomach were anastomosed to the jejunum in proper order. Two drains were used around the pancreaticojejunal and bilioenteric anastomosis.

Postoperative hemorrhage was defined as blood loss from drains or nasogastric tube with a drop of hemoglobin concentration of at least 3 g/dL. Time of hemorrhage after postoperative 24 hours was regarded as late hemorrhage according to the international definition. The locations of bleeding can be categorized into intraluminal, extraluminal, or both. Given that this study group did not contain early hemorrhage patients, we preferred to use 2 grades of postoperative hemorrhage (grade B and grade C) in accordance with the proposed classification by the International Study Group of Pancreatic Surgery.^[2]^ Grade B bleeding often demanded at least blood transfusion requirements, admission to an intensive care unit, and sometimes invasive therapeutic interventions such as transcatheter arterial embolization (TAE) and relaparotomy but without hemodynamic instability. On the contrary, grade C bleeding was usually life-threatening with an obvious decrease in patient mean arterial pressure under 90/60 mm Hg, which required immediate diagnostic and therapeutic consequences. Sentinel bleeding was defined as the occurrence of blood in the abdominal drainage tube or from the gastrointestinal tract without obvious cause, 24 hours before an episode of life-threatening massive bleeding. Patients receiving temporary endovascular treatment then underwent a subsequent laparotomy and were classified into a surgery group while analyzing management for the postoperative hemorrhage.

### 2.1 Definition of complications

In this study, intraabdominal erosive factors consisted of POPF, bile leakage, and abscess. POPF was defined as an amylase-rich drainage fluid (amylase level >3 times the upper limit of normal serum amylase concentration with any volume of drainage fluid) on or after postoperative day 3.^[6]^ Bile leakage was defined as the presence of bile in the abdominal drains with an increased bilirubin level of drainage fluid >3 times the serum bilirubin concentration measured at the same time on or after postoperative day 3.^[7]^ Intraabdominal abscess was defined by the presence of at least one of the following conditions: postoperative fever >38.5°C, abdominal pain, distension, and obvious peritoneal irritation signs; positive bacteriological culture results in the drainage fluid; or imaging studies confirming the presence of abdominal abscess. Postoperative bleeding after 24 hours following the index surgery without these erosive factors was defined as late nonerosive hemorrhage. On the contrary, late PPH with these erosive factors was classified into the erosive hemorrhage group.

### 2.2 Surgical procedure of LPD

The abdominal and liver surface was inspected to preclude tumor metastasis. The liver was suspended to the upper abdominal wall using hepatic needle by several transfixing sutures to facilitate future exposure of the hepatoduodenal structures. The greater omentum is transected along the greater curve longitudinally with an ultrasonic device; the Nos 4 and 6 lymph nodes were removed. The proximal jejunum approximately 3 cm distal to the Treitz ligament was exposed and divided through an avascular area in the transverse mesocolon on the left to the superior mesenteric vein. The pancreatic parenchyma was transected using an ultrasonic device; the pancreatic duct was divided by cold scissors 2 to 3 mm to the pancreatic remnant to facilitate future reconstruction. Along the superior border of the pancreas, the common hepatic artery (CHA) was found; the No. 8 lymph node was removed. Further dissection was performed along CHA and left gastric artery in the hepatoduodenal ligament; the Nos 5, 7, 9, and 12 lymph nodes were removed. After exposure of the left and right hepatic artery, the gastroduodenal artery was ligated with 2 clips. The common bile duct was exposed and divided with cold scissors; the upper resection margin would be sent for frozen section to preclude concomitant hilar cholangiocarcinoma if the primary lesion located at the distal common bile duct. The gallbladder was separated from the liver. Kocher maneuver was performed to mobilize the duodenal circle and expose the superior mesenteric vessels; the Nos 13, 14, and 16 lymph nodes were removed. Then the uncinate process can be identified and dissected from superior mesenteric vein. The specimen was removed through a 5-cm upper abdominal incision, which would be sealed by a rubber glove to maintain the pneumoperitoneum pressure during the following reconstruction. Reconstruction of digestive tract included pancreaticojejunostomy, hepaticojejunostomy, and gastrojejunostomy. Two drainage tubes were usually used with one above the bilioenteric anastomosis and another under the panreaticojejunal anastomosis. After surgery, the drainage tubes were checked daily for the character of drainage fluid and its volume. Amylase level in the drainage fluid was recorded on postoperative days 1 and 3. Five days after surgery, the drainage tubes were removed if the output volume was <50 mL and no pancreatic, other complication, or peritoneal effusion on computed tomography (CT) scan existed.

After LPD, patients received routine physiotherapy to prevent deep venous thrombosis of lower extremities such as gradient compression elastic socks. For patients who stayed in bed for more than 3 days without bloody drainage fluid, low molecular weight heparin calcium (4100 IU/d) was injected subcutaneously to prevent thrombosis.

### 2.3 Surgical procedure for external tube pancreatostomy

**Figure 1:**
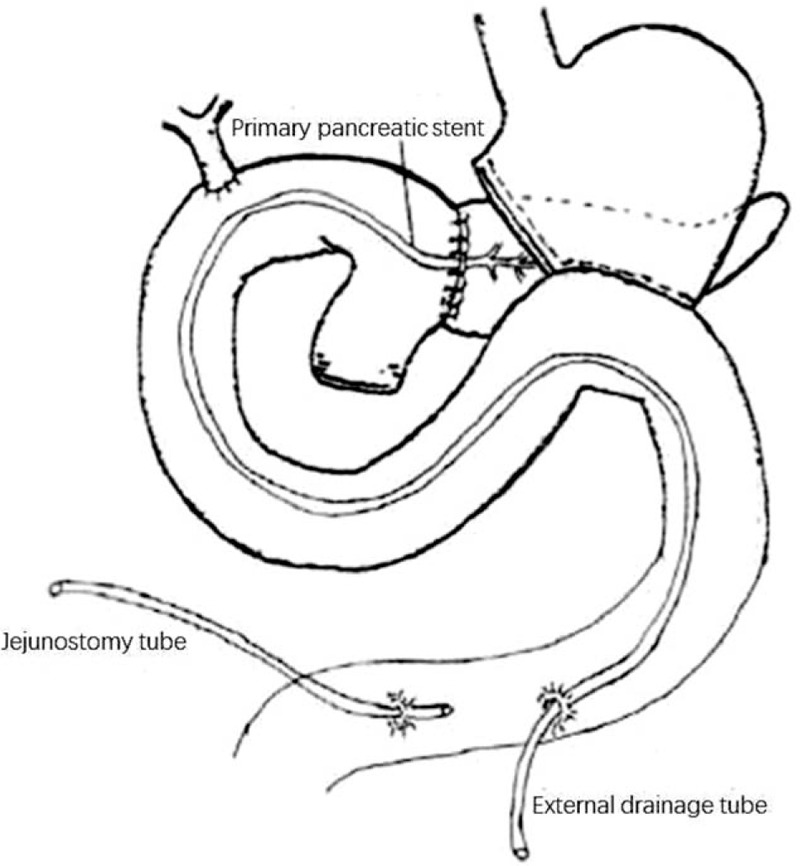
Surgical procedure for external tube pancreatostomy.

The original pancreaticojejunal anastomosis was opened and bleeding sites were controlled. Fine tube of appropriate caliber was selected and connected to the primary silicone tube in the pancreatic duct with some 5-0 sutures, though part of the jejunum, then extended out of the body. The original pancreaticojejunal anastomosis was fixed with simple sutures. Jejunostomy tube with 2 to 3 side holes was introduced into the distal jejunum 10 to 15 cm for future parenteral nutrition (Fig. 1).

### 2.4 Statistical analysis

Data were presented as mean ± standard deviation or median (interquartile range). The potential relationships among demographic factors, clinical characteristics, and mortality-related risk factors between the erosive and nonerosive groups were analyzed using the Yates corrected Chi-squared test or Fisher exact test for categorical variables. The Student *t* test was used to evaluate normally distributed variables with the Wilcoxon rank sum test for nonnormally distributed variables. *P* < .05 was regarded as statistically significant. All analyses were performed using SPSS, version 22.0 software.

## 3 Results

### 3.1 General characteristics of patients with erosive bleeding and nonerosive bleeding

The preoperative findings are given in Table 1. Of all 33 patients, 8 patients (24.24%) developed nonerosive bleeding and the other 25 patients (75.76%) suffered from postoperative hemorrhage caused by several reasons, such as pancreatic fistula, bile leakage, and intraabdominal abscess. There were 5 men and 3 women in the nonerosive group; their age varied from 33 to 74 years (mean 56.38 ± 15.11 years). The erosive bleeding group included 16 men and 9 women, and their ages ranged from 35 to 79 years (mean 60.16 ± 10.21 years). The patients with erosive bleeding tended to have higher body mass index (BMI) levels than the patients with nonerosive bleeding (24.06 ± 2.74 vs 22.19 ± 2.15 kg/m^2^) but without statistical significance (*P* = .089). Erosive and nonerosive bleeding patients had similar preoperative serum bilirubin and albumin levels (53.04 [17.02, 179.84] vs 39.3 [12.53, 139.38]) μmol/L, *P* = .401; and 38.62 ± 3.6 vs 39.08 ± 3.12 g/L, *P* = .754), respectively. However, an obvious trend that should be noted was that these 8 patients with nonerosive bleeding had no comorbidity (*P* = .004) and proposed no requirements for preoperative biliary drainage (*P* = .296).

**Table 1:**
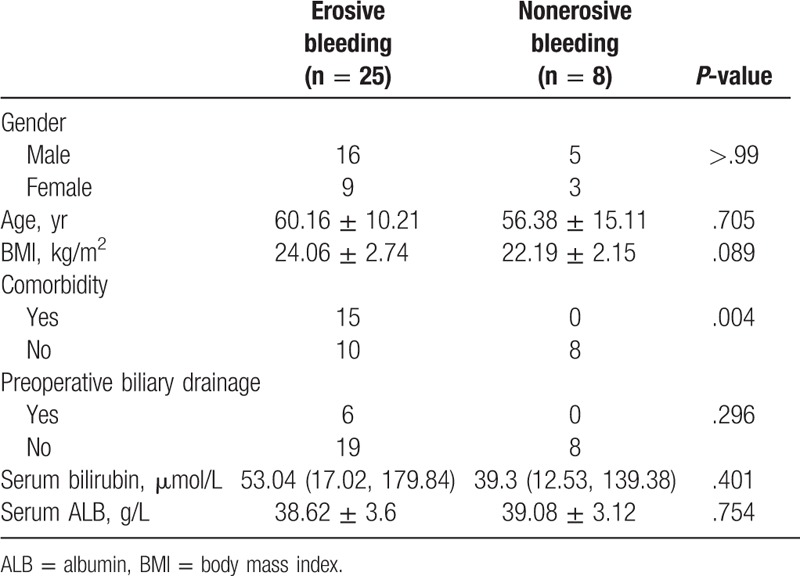
General characteristics of patients with erosive and nonerosive postpancreatectomy hemorrhage.

### 3.2 Clinical findings related to postoperative hemorrhage

**Figure 2:**
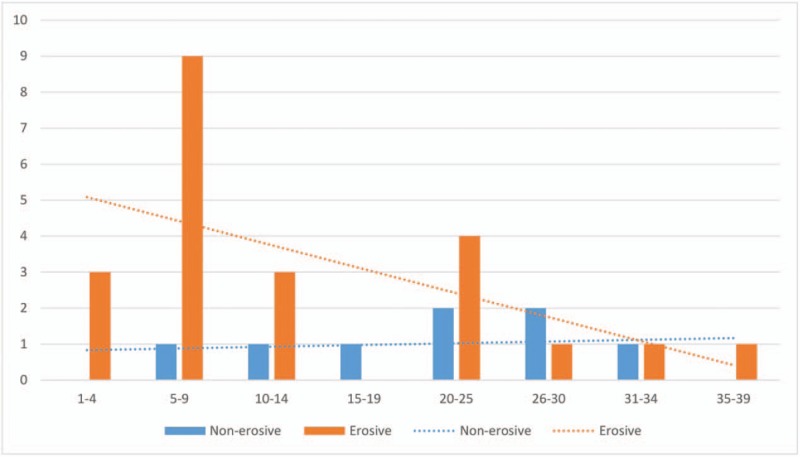
Time interval from laparoscopic pancreaticoduodenectomy to hemorrhage for erosive and nonerosive postpancreatectomy hemorrhage.

The clinical findings related to erosive and nonerosive PPH are provided in Table 2. The median interval from the LPD surgery to postoperative hemorrhage for both groups was 11 days, and no significant differences were found (*P* = .387). The flow diagram presenting bleeding intervals is provided in Figure 2. For patients with erosive bleeding, most (60%) underwent their episodes of bleeding on postoperative days 5 to 14. Two weeks after LPD surgery, the risk of postoperative bleeding decreased apparently among only 7 patients (28%) in the erosive bleeding group. For patients with nonerosive bleeding, most (75%) suffered from postoperative hemorrhage 2 weeks after surgery, and 50% of these patients had bleeding between postoperative days 20 and 30. Of note, both erosive and nonerosive bleeding patients could experience postoperative hemorrhage even 1 month after LPD.

**Table 2:**
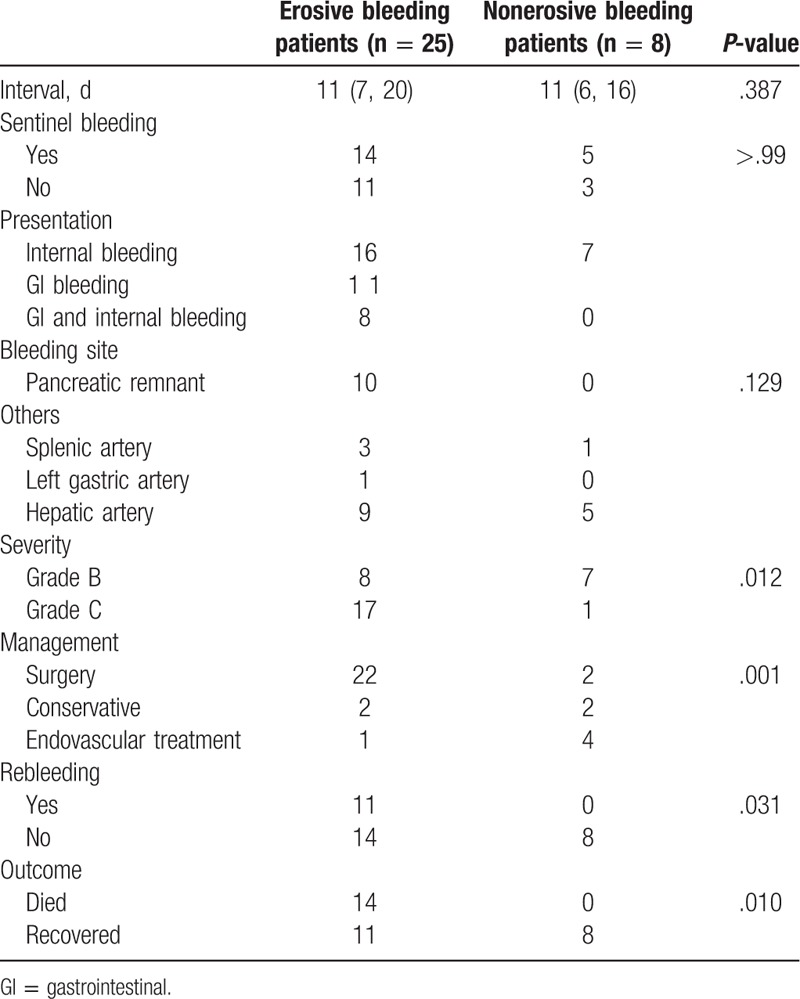
Clinical findings related to erosive and nonerosive postpancreatectomy hemorrhage.

**Figure 3:**
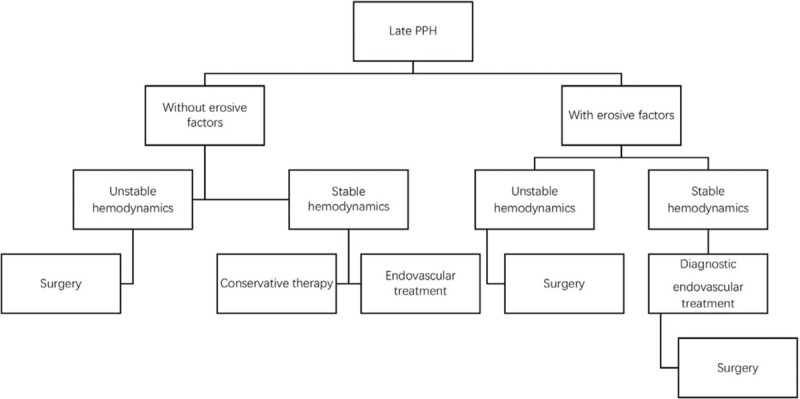
Conclusive treatment strategy for erosive and nonerosive postpancreatectomy hemorrhage (PPH).

**Figure 4:**
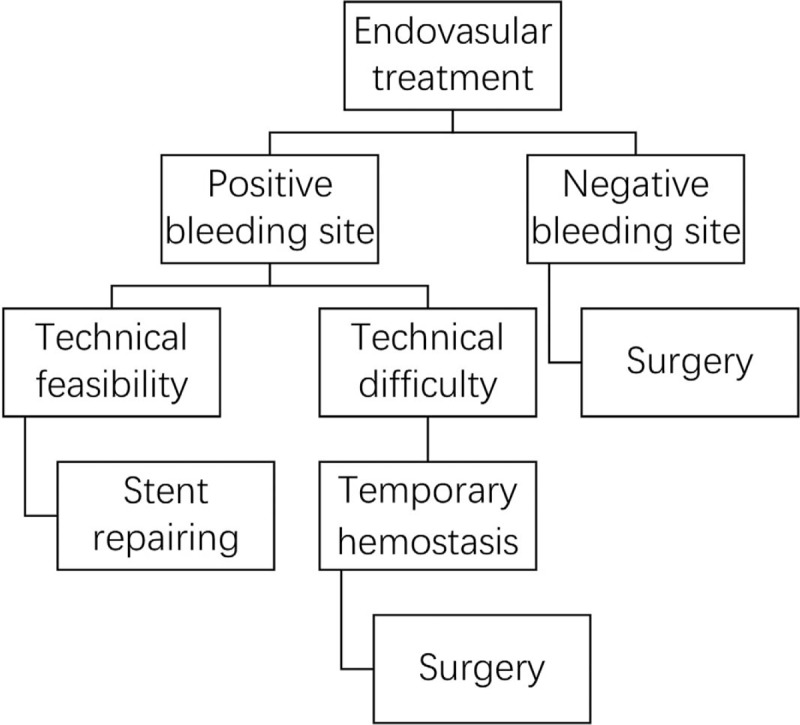
Practical indications for endovascular treatment with a covered stent.

Sentinel bleeding was found in 56% (14/25) of patients with erosive bleeding and 62.5% (5/8) of patients with nonerosive bleeding. In the present study, 64% (16/25) of patients with erosive bleeding, and 87.5% (7/8) of patients with nonerosive bleeding had internal bleeding. Combined internal and gastrointestinal bleeding occurred in 8 erosive bleeding patients, and only 2 patients (1 patient in each group) suffered from gastrointestinal bleeding alone. The fact that 90% (9/10) of all patients with gastrointestinal bleeding had intraabdominal erosive factors indicated strong associations between gastrointestinal hemorrhage and erosive factors. The bleeding sites were detected in most patients, except for 4 patients, who received conservative treatments. For patients with erosive bleeding, the most common bleeding site was the pancreatic remnant (43.48%); others included the hepatic artery (39.13%), splenic artery (13.04%), and left gastric artery (4.35%). For patients with nonerosive bleeding, the most common bleeding site was the hepatic artery (83.33%), and the 2nd most frequent site was the splenic artery (16.67%). No hemorrhage from pancreaticojejunal anastomosis occurred among the patients with nonerosive bleeding. Statistical significance was noted between these 2 groups in hemorrhage severity (*P* = .012), management strategies (*P* = .001), rebleeding occurrence (*P* = .031), and prognosis outcome (*P* = .010). The patients with intraabdominal erosive factors tended to have a higher risk of grade C bleeding (68.00%) than that of their nonerosive bleeding counterparts (12.50%). As for the treatment strategy for postoperative bleeding, the favorable method to manage nonerosive bleeding was conservative and endovascular treatments if the patients’ hemodynamics was stable. We performed emergency laparotomy in 2 patients with nonerosive bleeding (25.00%) and progressively dropping blood pressure. All 8 of these nonerosive bleeding patients survived. On the contrary, 22 patients (88.00%) in the erosive bleeding group had a 2nd surgery, and the mortality was 56.00%. In this group, 2 patients received conservative therapy due to the demand of their family and expired. One patient underwent endovascular treatment, and had another episode of hemorrhage, finally dying because of multi-organ failure. As for the occurrence of rebleeding, no patients in the nonerosive bleeding group suffered from rebleeding after complete hemostasis, and 44.00% of patients with erosive bleeding underwent a 2nd episode of postoperative bleeding. The conclusive treatment strategy for erosive and nonerosive PPH after LPD is summarized and provided in Figures 3 and 4.

According to hemodynamic stability, patients in the erosive and nonerosive bleeding groups were further analyzed. These results are provided in Table 3. In the nonerosive bleeding group, only 1 patient (12.50%) underwent an episode of internal hemorrhage with unstable hemodynamics and underwent an emergency laparotomy. Another patient undergoing a 2nd surgery had stable hemodynamics, and the decision that this patient received an emergency laparotomy directly, without angiography, was made according to the consideration and preference of the on-call doctor that night. In the erosive bleeding group, 68.00% (17/25) of patients suffered from severe postoperative hemorrhage with unstable hemodynamics. Higher incidences of gastrointestinal or gastrointestinal with internal bleeding (stable, 12.50%; unstable, 47.06%) rebleeding rate (stable, 25.00%; unstable, 52.94%), and mortality (stable, 37.50%; unstable, 64.71%) were observed in these 17 patients with unstable hemodynamics. All these results indicated a poor prognosis for patients suffering from erosive bleeding and unstable hemodynamics.

**Table 3:**
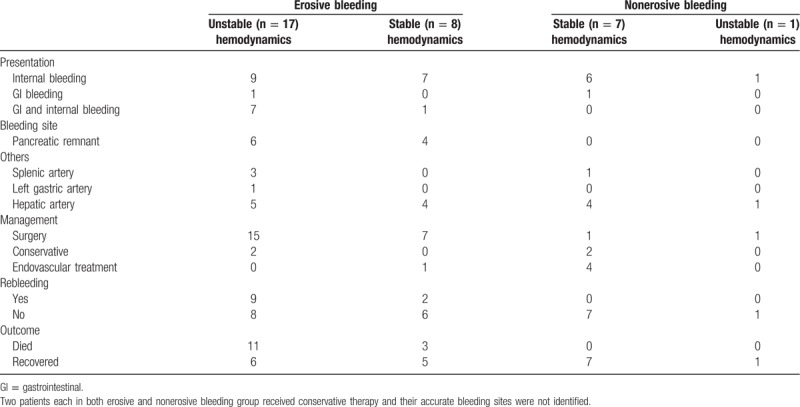
Subgroup analysis of erosive and nonerosive bleeding patients.

## 4 Discussion

The most enlightening effect of the current international definition of PPH timing is to differentiate early bleeding, usually caused by a technical failure of intraoperative hemostasis, from late bleeding due to several reasons, including pancreatic fistula, intraabdominal abscess, ulceration, and arterial pseudoaneurysm. Pancreatic fistula, bile leakage, and abdominal infection are generally recognized as risk factors of late PPH, which has been reported by several previous reports.^[3,8]^ Chen et al reported that 64.5% (40 out of 62 patients) of PPH patients were found to have a pancreatic fistula or abdominal infection, whereas this parameter declined to 25.5% in the no-PPH group. No accurate reasons could be found in those 22 (35.5%) patients in this study.

Different underlying etiologies may exist, because not all late PPH appears to be equal, so we proposed a novel definition of late PPH without erosive factors, including pancreatic fistula, bile leakage, and abdominal infection as nonerosive PPH. The reasons for this newly defined complication consist of ulceration at the site of anastomosis, small-vessel injury caused by intraabdominal drains, arterial pseudoaneurysm, and poor coagulation. According to the present study, a ruptured arterial pseudoaneurysm is the most common reason for late nonerosive PPH. The accurate mechanism of pseudoaneurysm formation in a nonerosive circumstance remains to be investigated. The possible explanation could be iatrogenic factors from surgical equipment causing endarterium damage during vascular skeletonization. These arterial injuries are difficult to find during the operation. After surgery, when the patient's blood pressure increased continuously, the pseudoaneurysm would gradually increase under the impact of the blood flow, oppressing the surrounding tissue, causing abdominal pain, and eventually rupturing. One patient undergoing a successful LPD always complained of abdominal pain after the index surgery, and a CT scan revealed nothing positive that could explain this pain. On postoperative day 17, this patient developed a sudden postoperative hemorrhage and the pain was relieved following the onset of intraabdominal bleeding. During endovascular treatment, this patient was found to have a ruptured pseudoaneurysm on the CHA and arterial dissection in the pseudoaneurysm, which caused the alternative pain.

Endovascular treatment has been reported to become the treatment of choice to control late PPH and achieve hemodynamic stabilization, especially for a ruptured pseudoaneurysm. In clinical practice, angiography is always necessary to identify the site of bleeding and cure patients experiencing PPH. The direct sign of bleeding in an angiography imaging is the overflow of a contrast agent when the velocity of bleeding is over 0.5 to 1 mL/min. Unsmooth vascular walls, vasospasm, and pseudoaneurysm formation are the indirect signs of a postoperative hemorrhage. The most commonly encountered cause of late PPH is a ruptured pseudoaneurysm. The total positive rate of angiography is 69% in identifying a hemorrhage after PD. The possible explanations for those false-negative cases could be that patients suffering from a massive bleeding cannot cooperate well with this examination because of hypoxemia, tachypnea, and hypovolemic shock, and the imaging would be relatively unclear. Another reason could be that no active contrast agent overflow was identified due to low velocity of the hemorrhage, under 0.5 to 1 mL/min at the intermittence of hemorrhage.^[9]^

The TAE always places micro-coils both proximally and distally to the bleeding site, so there are several negative reports regarding liver infarction after embolization of the proper hepatic artery.^[9]^ Fujii et al concluded an endovascular treatment strategy for managing postoperative hemorrhage after pancreaticobiliary surgery according to the site of bleeding and argued that the management should be individualized in accordance with the patient's own condition.^[10]^ The author suggested that TAE is the treatment of choice for bleeding located at the splenic artery and distal to the proper hepatic artery (right hepatic artery, middle hepatic artery, left hepatic artery), and highly selective TAE should be performed to preserve the blood flow of other branches to the liver. However, the successful implementation of this technically demanding procedure depends on the expertise of the operator, and inadvertent occlusion of the proper hepatic artery may occur, causing fatal liver infarction. As for bleeding sites located proximally to the proper hepatic artery (gastroduodenal artery, CHA, celiac artery), TAE is associated with a discouraging outcome and not recommended unless there is a replaced hepatic artery or well-developed subphrenic artery. If the answer to this question is negative, endovascular stenting is a favorable solution in this situation, interrupting the hemorrhage and preserving the patency of blood vessels. However, a major problem with this type of classification is that surgeons have resected the gastroduodenal artery during the PD procedure so that proximal or distal locations to the proper hepatic artery are technically similar. Therefore, we cannot apply this classification system to the patients undergoing LPD at our department. At our department, we prefer not to use TAE, considering the highly possible rebleeding due to incomplete embolization, coil compression, and migration.

Covered stents have evolved as new applications to treat postoperative hemorrhages. In 1998, McGraw et al reported the 1st successful use of this device to manage a ruptured pseudoaneurysm from the superior mesenteric artery. In 2000, Burger et al reported the 1st successful repair of a hepatic artery aneurysm following PD. The most obvious advantage of stent repairing is the preservation of arterial blood flow.^[11]^ Given that the stent-repairing technique is theoretically superior to arterial embolization, we should consider these hemorrhage conditions as 2 types: positive bleeding site; negative bleeding site. If the bleeding site is easy to discover, stent repairing should be used without hesitation. When this procedure is technically difficult or the patient's hemodynamics continuously worsens, emergency laparotomy should be considered after temporary hemostasis by arterial embolization occluding the vessel proximal to the bleeding site. If the bleeding site is difficult to discover, an emergency laparotomy may be a better choice to control bleeding.

In the setting of a patient's stable hemodynamics, the efficacy of endovascular treatment to find bleeding points is remarkable and distinguished. When there are no erosive factors in the patients’ abdominal cavity, this procedure can be used to achieve continuous hemostasis and save surgeons from a technically demanding emergency relaparotomy. In the other situation, with erosive factors in the patients’ abdominal cavity, endovascular treatment still can be used to identify the bleeding sites and obtain temporary hemostasis but it is not a reliable method to eradicate rebleeding secondary to the intraabdominal erosive factors. Thus, we prefer to perform endovascular treatment to find the bleeding sites and release a covered stent to stop bleeding, and then implement a relaparotomy to dissolve any primary causes such as pancreatic fistula.

Although endovascular treatment has been developed recently and is considered a safe and effective procedure to control late hemorrhage after PD, the surgical approach should be adopted under the following conditions: severe active bleeding endangering hemodynamic stability; failure of endovascular or endoscopic treatment; bleeding from venous systems such as the portal vein and its tributaries; persistent intraabdominal erosive factors such as pancreatic fistula, abscess, or bile leakage. Endovascular treatment for late PPH cannot solve the crucial problem of the intraabdominal erosive factors causing the life-threatening complication. Surgery is the only option to control bleeding and manage the primary causes simultaneously, and rebleeding after arterial embolization is considered a poor prognostic factor. Zhou et al reported a rebleeding rate of 17.2% for all bleeding patients after angiography and endovascular treatment.^[9]^ In this study, rebleeding occurred in the patient with intraabdominal erosive factors and receiving endovascular treatment but not a patient in the nonerosive group. Rebleeding is often secondary to intraabdominal erosive factors that demand surgical treatments. Hence, we do not recommend this therapeutic option as an independent 1st-line treatment for patients with erosive bleeding. The reason may be that this procedure still risks rupturing vessels because of the fragile vascular wall due to the intraabdominal erosive condition.

Relaparotomy for PPH is technically difficult, considering the following conditions: extensive adhesion in the upper abdomen caused by the strong corrosion ability of pancreatic juice; limited length of the afferent limb for resection and reconstruction due to the fixed location of bilioenteric and gastrointestinal anastomosis. Several surgeons attempted to remove the former pancreaticojejunal anastomosis and perform a pancreaticogastrostomy reconstruction. However, despite the difficulty of implementing this procedure in an emergency operation, the pancreaticogastrostomy anastomosis is considered an independent risk factor for postoperative hemorrhage after PD.^[12]^ We recommend external tube pancreatostomy (ETP) as the treatment of choice for the patients with late PPH and intraabdominal erosive factors. This procedure can effectively drain the pancreatic juice, prevent its continuous corrosion of the surrounding tissue, and prevent the pancreatic enzymes from activating. Compared to completion pancreatectomy (CP), the prominent advantage of this procedure lies in its simplicity: surgeons do not have to dissect the pancreaticojejunal anastomosis and adjacent areas extensively. When rebuilding the pancreaticojejunal anastomosis, it demands only simple connections between the former pancreatic stump and the jejunum, which is not difficult to accomplish even if there is tissue edema. Although there are risks of bile or intestinal leakage through this rough pancreaticojejunal anastomosis, given no pancreatic enzyme activation, these complications are usually cured by conservative treatment such as continuous drainage and perfusion. Meanwhile, these patients always received a jejunostomy tube and early enteral nutrition to promote intestinal peristalsis, shorten the time of fasting, and reduce intestinal bacterial translocation and infection.

Emergency CP carries a high risk of postoperative morbidity and mortality compared to ETP and should be reserved as a final therapeutic choice for highly selected patients with a complicated pancreatic fistula and necrosis.^[13]^ Compared to CP, this pancreas-preserving technique has several other theoretical advantages: it is an easier procedure, causes less blood loss, and requires shorter operative time. Balzano et al studied the clinical data of 14 CP patients and reported that the duration of relaparotomy and estimated blood loss were 240 ± 72 minutes and 2507 ± 1976 mL, respectively.^[14]^ In a retrospective study analyzing data, including 136 CP patients (elective CP, n = 98; emergency CP, n = 38) between 1987 and 2013, Almond et al found that the relative use of emergency CP is decreasing by 0.28 percentage points annually; the postoperative complication rate for emergency CP was up to 78.95% (30/38), and major morbidity included delayed gastric emptying, bile leak, and hemorrhage.^[15]^ This high morbidity indicates poor outcomes following salvage CP when severe intraabdominal infection or massive bleeding after PD exists. However, the greatest disadvantage of CP is the inevitable occurrence of brittle diabetes when compared to ETP. The life quality of patients undergoing CP has been reported to be quite similar to those patients with diabetes caused by other reasons, so surgeons should try to avoid this procedure, especially in patients with a long life expectancy and benign disease. Hence, given the technical challenge and poor prognosis attached to emergency CP, we recommend CP as a final salvage procedure for the patients in the setting of serious anastomotic dehiscence and necrosis causing the infeasibility to find the pancreatic duct.

The endoscope has been considered to control hemorrhage after abdominal surgery, especially in stress ulcer bleeding. The obvious advantage of this treatment method is that the endoscope can manage this problem at the time of diagnosis. However, endoscopic hemostasis for PPH was not used in the treatment protocol in our department, considering the following conditions. The 1st disadvantage of gastroscopy is that massive and active bleeding may blur the vision and prevent surgeons from finding the bleeding source. The 2nd disadvantage is the poor access of the endoscope to the pancreaticojejunal and bilioenteric anastomosis. The third disadvantage is the false gastrointestinal hemorrhage caused by intraabdominal pancreatic remnant bleeding.

To prevent the formation of an arterial pseudoaneurysm effectively, we have summarized our experiences with more than 440 LPDs and proposed the following suggestions: While manipulating the abdominal organs, surgeons should avoid pulling the clipped blood vessels to decrease the risk of rebleeding of vascular remnants and reduce the endarterium damage. The stump of the gastroduodenal artery is always considered a high-risk site of postoperative pseudoaneurysm formation, and complete skeletonization of this area should be avoided. When clipping arteries with biological clips, the motions should be slow and steady to protect the vessels from cutting injuries. The work arm of the ultrasonic device in the motivated state cannot touch the blood vessels directly. After the reconstruction of the gastrointestinal tract, the greater omentum can be used to cover the skeletal vessels to form an effective protective package and prevent postoperative hemorrhage.

For late PPH, we classified these patients into 2 groups according to the existence of abdominal erosive factors or not and proposed a treatment protocol in the present study. For patients with erosive factors, surgery would be the 1st choice to control bleeding, given the primary causes of hemorrhage (erosive factors). When the patients’ hemodynamics is stable, endovascular treatment should be considered as both a diagnostic and temporary therapeutic method. As for the procedure for relaparotomy, compared to CP, we prefer ETP as the 1st-line surgical procedure, mainly due to its technical simplicity, reduced morbidity, and preservation of pancreatic function. CP should be considered in the setting of serious anastomotic dehiscence and necrosis, causing inability to find the pancreatic duct. As for nonerosive bleeding, when severe active bleeding endangers the patient's hemodynamic stability, emergency relaparotomy should be performed without any hesitation, and a simple suture would manage the bleeding site successfully, given no erosive factors. When the patient's hemodynamics is stable, multiple methods could be adopted, such as conservative therapy and endovascular treatment. Conservative therapy and intensive observation could be used in the setting of minor venous bleeding, whereas endovascular treatment should be considered if surgeons suspect active arterial bleeding.

Endovascular treatment for managing postoperative hemorrhage after PD varies according to the discovery of the bleeding site. We should consider these hemorrhage conditions as 2 types: positive bleeding site; negative bleeding site. If the bleeding site is easy to discover, stent repair should be used without hesitation. When this procedure is technically difficult or the patient's hemodynamics continuously worsens, emergency laparotomy should be considered after temporary hemostasis by arterial embolization occluding the vessel proximal to the bleeding site. If the bleeding site is difficult to discover, an emergency laparotomy may be a better choice to control bleeding. Of note, angiography is highly recommended to detect the site of bleeding, but surgery should not be excessively delayed.

## 5 Conclusion

The PPH remains a rare but lethal complication following LPD in the modern era of advanced surgical techniques. According to the existence of intraabdominal erosive factors, we classify the patients with postoperative bleeding after LPD into erosive and nonerosive bleeding groups. Their severity of bleeding, rebleeding rate, and treatment strategy are different. Patients with erosive factors tend to have a higher incidence of grade C bleeding, rebleeding, and mortality. Factors influencing treatment protocols for PPH include the existence of intraabdominal erosive factors, patient hemodynamics, possibility to detect the bleeding site during endovascular treatment, and surgeon's preference. The performance of endovascular treatment with stent repair for managing postoperative hemorrhage after LPD depends on the discovery of the bleeding site. Surgery should be reserved as an emergent and final choice to manage PPH.

## Author contributions

**Formal analysis:** Xuehui Cao.

**Investigation:** Xuehui Cao.

**Resources:** Xueqing Liu.

**Software:** Jianzhang Qin.

**Methodology:** Zhongqiang Xing.

**Data curation:** Jiayue Duan.

**Validation:** Chen Liu.

**Writing – original draft:** Feng Feng.

**Writing – review & editing:** Jianhua Liu.
